# Recurrence Quantification Analysis of Ankle Kinematics During Gait in Individuals With Chronic Ankle Instability

**DOI:** 10.3389/fspor.2022.893745

**Published:** 2022-05-25

**Authors:** Sheng-Che Yen, Shaodi Qian, Eric Folmar, Christopher J. Hasson, Chun-An Chou

**Affiliations:** ^1^Department of Physical Therapy, Movement and Rehabilitation Sciences, Bouvé College of Health Sciences, Northeastern University, Boston, MA, United States; ^2^Department of Mechanical and Industrial Engineering, College of Engineering, Northeastern University, Boston, MA, United States; ^3^Department of Bioengineering, Northeastern University, Boston, MA, United States; ^4^Department of Biology, Northeastern University, Boston, MA, United States

**Keywords:** functional instability, complexity, walking, recurrence plot (RP), laminarity (LAM), determinism (DET)

## Abstract

**Purpose:**

An investigation of the ankle dynamics in a motor task may generate insights into the etiology of chronic ankle instability (CAI). This study presents a novel application of recurrence quantification analysis (RQA) to examine the ankle dynamics during walking. We hypothesized that CAI is associated with changes in the ankle dynamics as assessed by measures of determinism and laminarity using RQA.

**Methods:**

We recorded and analyzed the ankle position trajectories in the frontal and sagittal planes from 12 participants with CAI and 12 healthy controls during treadmill walking. We used time-delay embedding to reconstruct the position trajectories to a phase space that represents the states of the ankle dynamics. Based on the phase space trajectory, a recurrence plot was constructed and two RQA variables, the percent determinism (%DET) and the percent laminarity (%LAM), were derived from the recurrence plot to quantify the ankle dynamics.

**Results:**

In the frontal plane, the %LAM in the CAI group was significantly lower than that in the control group (*p* < 0.05. effect size = 0.86). This indicated that the ankle dynamics in individuals with CAI is less likely to remain in the same state. No significant results were found in the %DET or in the sagittal plane.

**Conclusion:**

A lower frontal-plane %LAM may reflect more frequent switching between different patterns of neuromuscular control states due to the instabilities associated with CAI. With further study and development, %LAM may have the potential to become a useful biomarker for CAI.

## Introduction

A lateral ankle sprain is one of the most prevalent orthopedic and sports injuries that can lead to long-term complications (Waterman et al., 2010; Wallace et al., 2011). Following an initial sprain, many individuals experience lingering residual symptoms such as frequent episodes of ankle giving way and recurrent ankle sprains (Hertel, 2002). These residual symptoms are often referred to as chronic ankle instability (CAI). CAI has been linked to several negative health-related outcomes such as a decrease in the physical activity level (Hubbard-Turner and Turner, 2015) and an increase in the likelihood to develop posttraumatic osteoarthritis (Hintermann et al., 2002). While significant research effort has been made on understanding the etiology of CAI, the contributing factors to this medical problem remain unclear. However, it is widely accepted that the cause of CAI involves not only mechanical problems (joint laxity) but also sensorimotor control problems (Hertel and Corbett, 2019).

The ankle joint plays an important role in gait, and therefore many studies examined if CAI is associated with changes in ankle kinematics during gait (Delahunt et al., 2006; Monaghan et al., 2006; Chinn et al., 2013; Herb et al., 2014). Collectively, these studies showed that individuals with CAI tend to walk with increased ankle inversion and plantarflexion compared to their healthy counterparts (Moisan et al., 2017). These kinematic changes place the ankle in a more open-packed position and reduce the mechanical stability of the ankle. In addition, individuals with an overly inverted ankle are more likely to land on the lateral side of the foot during initial contact, which increases the risk of lateral ankle sprains.

In addition to changes in kinematic patterns during gait, researchers have also examined whether CAI is associated with changes in gait variability. Variability is inherent within all biological systems, and naturally, there are stride-to-stride fluctuations when people walk or run. Studying variability may generate insights into the sensorimotor process of a dynamical system. For example, variability may indicate errors in motor execution (Schmidt, 2003) or the ability to adapt to the environment or task changes (Kelso, 1995). In addition, neuromuscular or musculoskeletal injuries may be associated with an increase or decrease in movement variability (Stergiou et al., 2006; Stergiou and Decker, 2011; Hamill et al., 2012).

Previous studies examining changes in gait variability associated with CAI often used linear tools to quantify the variability (Drewes et al., 2009; Herb et al., 2014; Kautzky et al., 2015). Linear methods, such as standard deviation, quantify how each movement deviates away from the “mean” movement pattern and measure the “magnitude” of variability. However, linear methods do not quantify the structural nature of this variability, e.g., the existence of patterns or temporal regularities in the behavior of a system generating the movements. For example, the standard deviation of a hypothetical time series {2,3,4,2,3,4} is the same as the standard deviation of another time series {2,4,3,3,4,2}. However, the former time series shows a greater degree of temporal regularity (or repeatability) compared to the latter one. This example shows that linear methods cannot detect the variation in temporal sequence.

These limitations can be addressed using nonlinear methods such as sample entropy (Stergiou and Decker, 2011). These methods can capture the time-evolving behavior of a complex adaptable system, i.e., a system which may be sensitive to initial conditions adapts its behavior using feedback, and is influenced by its environment in a nonlinear way (Johnson, 2011). A previous study used sample entropy to compare the complexity of ankle kinematics during walking between individuals with and without CAI (Terada et al., 2015). The same technique was used to compare the complexity of the center of pressure trajectories during a single-leg balance task between those with and without CAI (Terada et al., 2019). Complexity refers to the predictability of a system's behavior (e.g., a data series with low complexity may have a pattern that makes it easy to predict future states, such as a sine wave) (Delgado-Bonal and Marshak, 2019). However, the sample entropy technique was applied to the original ankle position trajectory, which was a one-dimensional time series (variation of range of motion over time) that may not depict all critical information regarding the ankle dynamics.

In this study, we present a novel application of recurrence quantification analysis (RQA) (Webber and Zbilut, 2005) to analyze ankle dynamics in individuals with CAI. RQA is a nonlinear method that uses time-delay embedding to reconstruct a chaotic dynamical system in higher-dimension phase space. The phase space trajectory is then converted to a recurrence plot from which several RQA variables, such as determinism and laminarity, can be computed to characterize different aspects of system dynamics (Marwan et al., 2007). Determinism indicates how often the phase space trajectory repeats itself over time and shows how predictable the dynamic system is. Laminarity indicates how often the phase space trajectory stays in a specific state. These variables may provide access to information about ankle dynamics that is not observable in a compressed one-dimensional signal. They have the potential to be developed as useful clinical tools to evaluate gait and posture in individuals with sensorimotor control deficits. For example, a previous study examined ground reaction force patterns during gait using RQA to differentiate patients with Parkinson's disease in different stages of progression (Afsar et al., 2018). Another study used RQA to examine age-related changes in postural stability (Li et al., 2018). This current study was an initial effort to understand if RQA can differentiate individuals with or without CAI based on their ankle kinematics during gait.

The purpose of this study was to compare ankle dynamics during walking between individuals with and without CAI using RQA. We hypothesized that CAI is associated with changes in the ankle dynamics as assessed by measures of determinism and laminarity using RQA.

## Methods

### Participants

A total of twelve participants with CAI (age = 23.7 ± 2 years old; body mass index or BMI = 23 ± 1.8; two males and 10 females) and 12 gender- and age-matched (within 2 years of difference) healthy controls (age = 24.5 ± 1.6 years old; BMI = 21.6 ± 1.6) were conveniently recruited from a university campus in 2016 and 2017. More females responded to our recruitment than males. All participants with CAI had an initial ankle sprain that occurred 1 year before enrollment, had multiple episodes of ankle giving way in the past 6 months before enrollment, and scored 24 or lower in the Cumberland Ankle Instability Tool (CAIT) (Hiller et al., 2006). All healthy controls had a CAIT score equal or above 28, with no previous history of an ankle sprain. Potential participants were excluded from the study if they had a history of fracture or surgery in either lower limb. All participants signed an informed consent approved by the local institutional review board before participating in the study.

### Data Collection Procedures

Participants were asked to walk with their sneakers at a self-selected comfortable speed on a treadmill for 1 min. A warm-up trial was provided before testing so that participants were able to familiarize themselves with walking on a treadmill. Researchers determined each participant's self-selected comfortable speed during the warm-up trial based on his/her self-report. The two groups had similar comfortable walking speeds (CAI group: 1.16 ± 0 m/s; control group: 1.15 ± 0.27 m/s). Participants' ankle position change over time was captured using a 3D motion capture system (Qualisys AB, Göteborg, Sweden). The sampling frequency was set at 100 Hz. The marker placement and procedures for creating an ankle model were based on our previous study (Yen et al., 2015) and were consistent with the guidelines provided by Visual3D (C-Motion Inc, Rockville, MD). Briefly, reflective markers were placed on the following bony landmarks to create shank and foot models: medial and lateral femoral epicondyles, medial and lateral malleoli, and the second and the fifth metatarsal heads. A rigid cluster of four markers placed on the posterior shank was used to track the segment. The marker on the second metatarsal head and two additional markers placed on the posterior and the lateral sides of the calcaneus were used to track the foot segment.

For each participant, we analyzed the ankle motion trajectory of the affected leg (CAI participant) or the matched leg (healthy control) in the sagittal plane (dorsi- and plantarflexion) and frontal plane (eversion and inversion) over 25 consecutive strides using RQA. For CAI participants who had bilateral instability, the more severe side (based on the CAIT score) was selected for analysis. A stride was defined as the period between two consecutive initial contacts of the same foot. Initial contact was determined as the time when the marker on the calcaneus changed its moving direction from forward to backward (Zeni et al., 2008). Each participant's trajectory was scaled to 2,500 sample points before the analysis. Although RQA can be computed for any length of data, Brick et al. (2018) suggest a minimum of three or four measurements within each repetition of a pattern of interest. Our 100 Hz sampling rate would give 10 samples in a single cycle of a 10 Hz signal. During gait, most of the power in the frequency spectrum is below 10 Hz (Angeloni et al., 1994), and thus we exceed these requirements by at least a factor of two. One participant only had 24 consecutive strides due to a technical issue during data collection. Based on our preliminary analysis, removing this participant had minimal impact on the results, and therefore we included this participant in the report.

### Phase Space, Recurrence Plot, and RQA Variables

**Figure 1:**
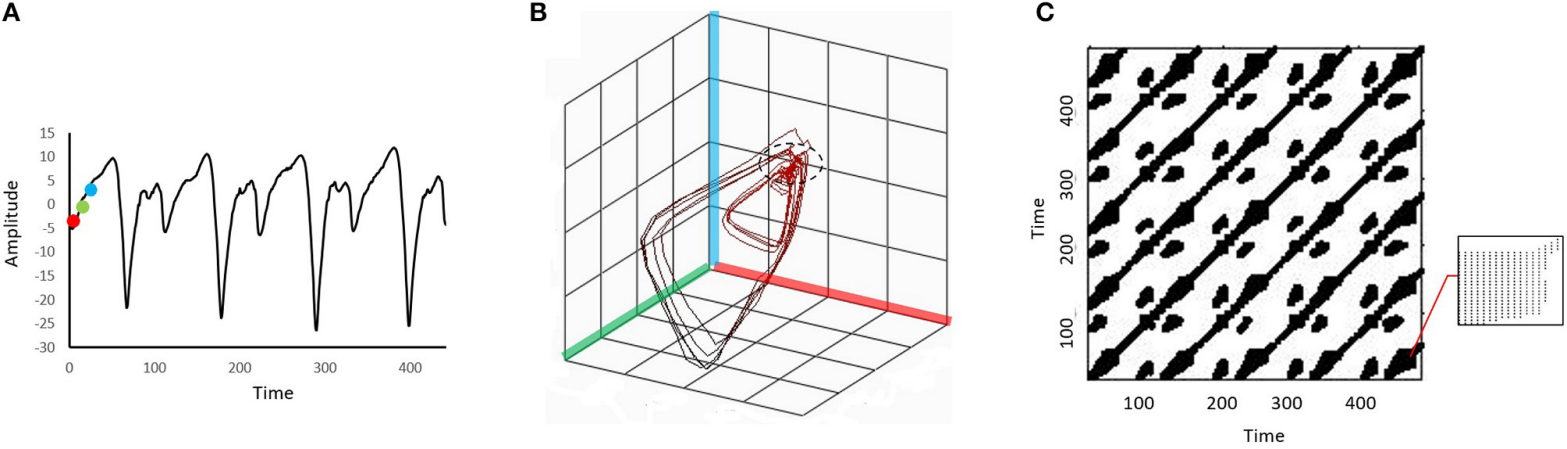
An illustration of the procedures to convert a time series to a 3-dimensional phase space before it is projected to a recurrence plot. A point (red dot) and two other points in a later time (green and blue dots) are identified in the original time series **(A)**. These three dots are then embedded in a 3-dimensional phase space formed by the red, green, and blue axes **(B)**. Each location in the phase space is viewed as a “state” of the dynamic system. The same embedding procedure is repeated for every point on the original time series, and a trajectory will be formed in the phase space. The phase space trajectory is then projected to the recurrence plot **(C)**. The recurrence plot is essentially an N-by-N grid, where N is the number of points in the original time series. The black dots on the grid represent recurrence points. When the system repeats specific sequences of stats in the phase space (i.e., the “pattern” of the phase space trajectory repeats), recurrence points will form diagonal lines of the recurrence plot. When the system stays in a similar state for a period (e.g., the part of the phase trajectory marked by the dashed circle in **(B)**), recurrence points will form vertical lines. The bottom right corner of the recurrence plot **(C)** is zoomed in to show the vertical lines. In this example the embedding dimension was set artificially low and a relatively large distance threshold was used to facilitate conceptual understanding of the structures in the recurrence plot.

We performed RQA following the procedures described by Webber and Zbilut (2005). Figure 1 illustrates the procedures using a simplified example for visualization purposes. The parameters used to construct Figure 1 were different from the parameters used in the actual analysis.

In the first stage of RQA, the ankle position trajectory was reconstructed to a phase space with time-delay embedding. Specifically, the position trajectory was embedded in a 5-dimension phase space with a time delay of 10 samples. The dimension number was set according to the false nearest neighbors' method, and the time delay was set based on the detection of the first local minimum of the mutual information (Zbilut and Webber, 1992). In principle, the phase space contains all possible states of the ankle dynamics, and each state corresponds to a unique location in the phase space.

In the second stage, recurrence points were identified from the phase space trajectory. Points on the phase space trajectory were considered recurrent when the Euclidean distance between them was below a predetermined distance threshold (i.e., these points were in a similar location or state). In this study, the distance threshold was set at 10% of the mean distance of all pairwise points (Schinkel et al., 2008). In the last stage, the recurrence points were projected to a 2-dimensional recurrence plot (RP). The RP is essentially an N-by-N grid, where N is the number of points in the original time series. The black dots on the grid represent recurrence points.

Two RQA variables, the percent determinism (%DET) and the percent laminarity (%LAM), were derived from the RP to quantify the ankle dynamics. The formulas used to calculate these two variables were presented in Marwan et al. (2007). Briefly, the %DET is the percentage of recurrent points that forms the diagonal lines in the RP. It indicates how often the system repeats specific sequences of stats when moving in the phase space (i.e., the pattern of the phase space trajectory repeats itself). A higher %DET indicates that the system is more predictable. The %LAM measures the percentage of recurrent points that forms the vertical lines in the RP. It indicates how often the phase space trajectory remains (or is “trapped”) in a specific state. The state where the phase trajectory stays for a period is called a laminar state (Marwan et al., 2007). A higher %LAM indicates that the system is less likely to switch between different states. Figure 1 shows the illustration of diagonal and vertical lines.

### Statistical Analysis

Each RQA variable was compared between the CAI and control groups using independent *t*-tests. Before carrying out the *t*-tests, its underlying assumptions of normality and homogeneity of variance were tested using the Shapiro–Wilk's test and Leven's test, respectively. We found all these assumptions were held. All statistical tests were performed using SPSS v25 (IBM, NY). The alpha level was set at 0.05. In addition, Cohen's d was calculated to determine the effect size of the results, with d = 0.2 indicating a small effect, 0.5 a medium effect, and 0.8 a large effect (Cohen, 2013).

## Results

### Recurrence Plots

**Figure 2:**
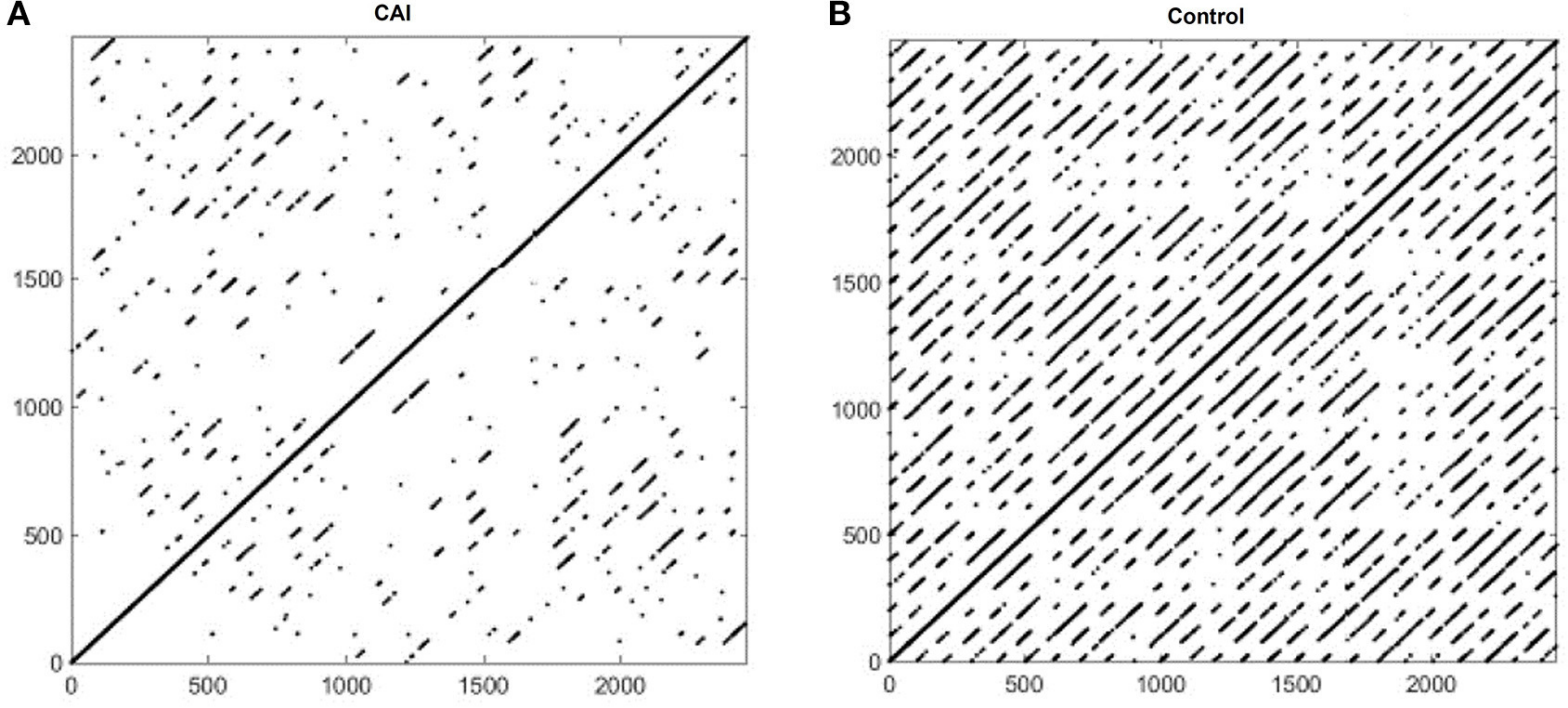
Recurrence plots of an example participant with CAI **(A)** and healthy control **(B)** in the frontal plane. CAI, chronic ankle instability.

**Figure 3:**
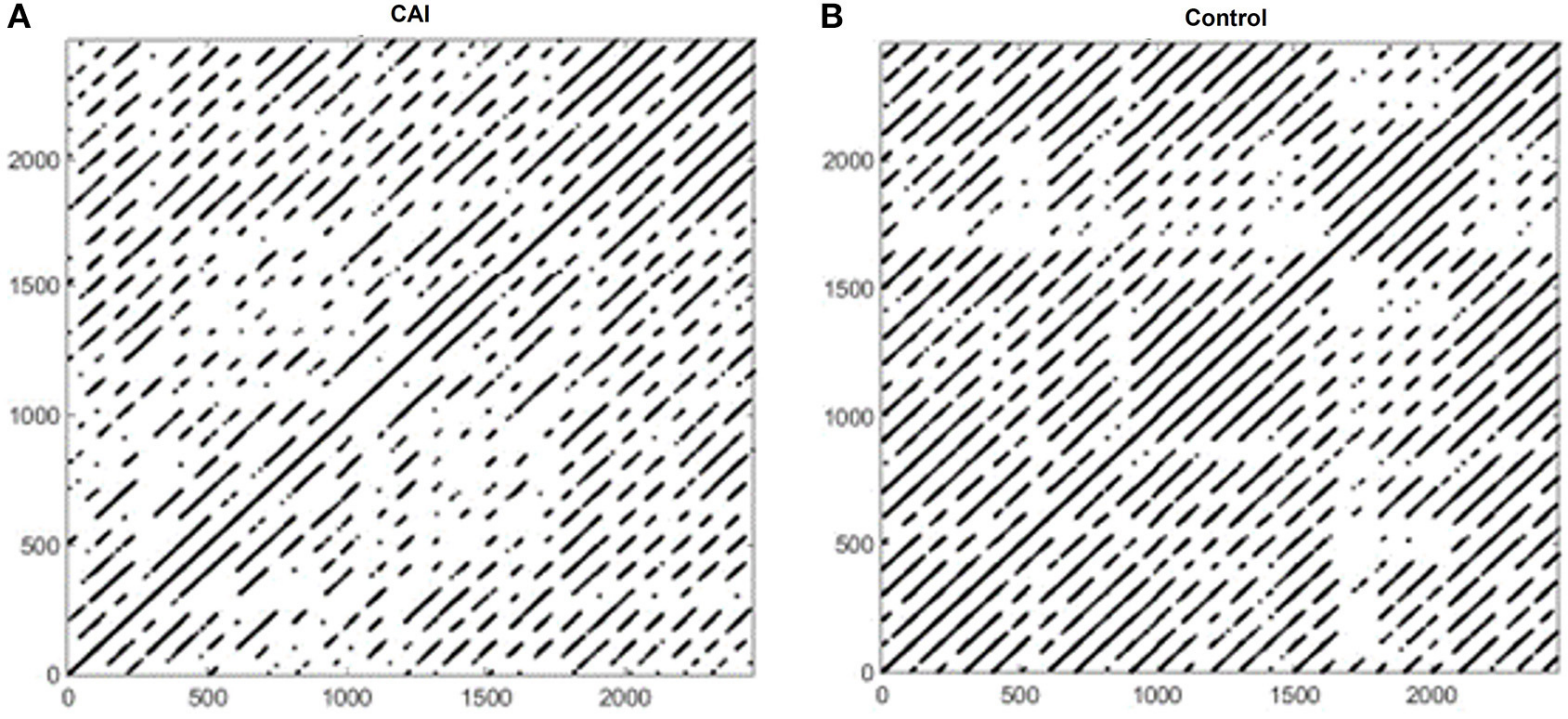
Recurrence plots of an example participant with CAI **(A)** and healthy control **(B)** in the sagittal plane. CAI, chronic ankle instability.

Figure 2 shows example RPs in the frontal plane from a participant with CAI (Figure 2A) and healthy control (Figure 2B). There are fewer recurrence points (black dots) in the participant with CAI compared to the control. In addition, the percentage of recurrence points on the diagonal lines (%DET) and on the vertical lines (%LAM) is less in both the CAI participants. Compared to the RPs in the frontal plane, the RPs in the sagittal plane (Figure 3) contain a higher density of recurrence points, regardless of the ankle condition. In addition, the percentage of recurrence points on the diagonal lines and the vertical lines are both similar between the example participants with and without CAI (Figures 3A,B).

### RQA Variables

**Figure 4:**
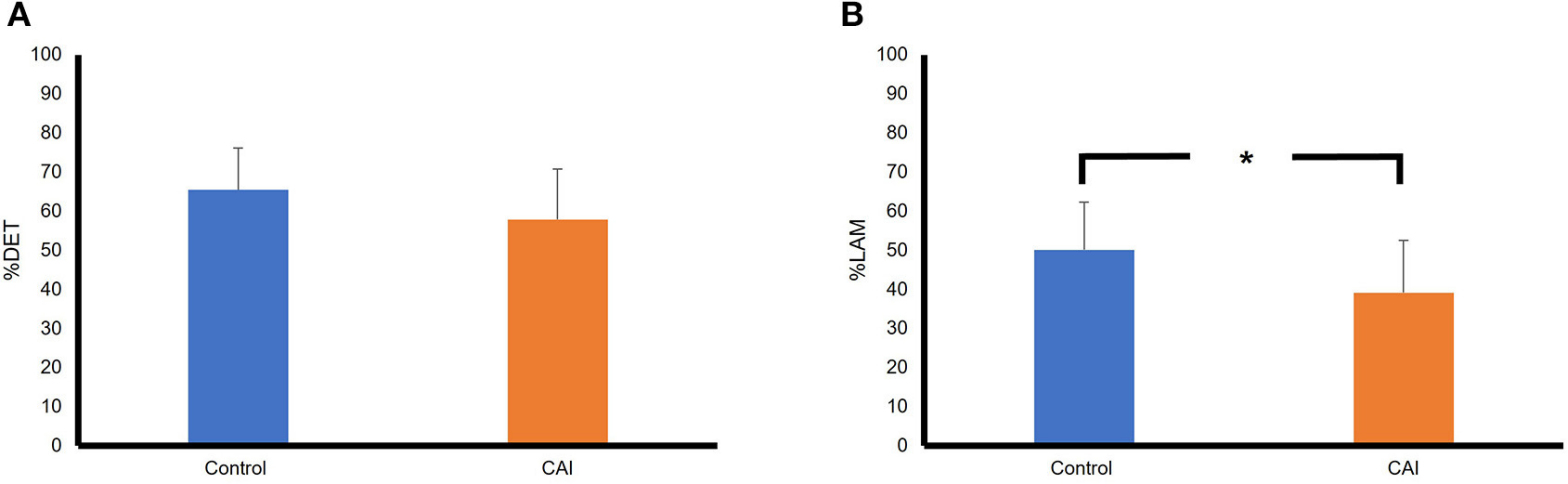
Comparisons of RQA variables between the CAI and control groups in the frontal plane: **(A)** A comparison of percent determinism (%DET); **(B)** A comparison of percent laminarity (%LAM). The error bar represents the standard deviation. *p < 0.05. RQA, recurrence quantification analysis; CAI, chronic ankle instability.

Figure 4 summarizes the results of RQA variables in the frontal plane. Based on the mean, the %DET was higher in the control group than that in the CAI group, although the difference did not reach a statistical significance (Figure 2A), t(22) = 1.53, *p* = 0.14, Cohen's d = 0.58, a moderate effect. The %LAM was also higher in the control group than that in the CAI group, and the difference reached statistical significance (Figure 2B), t(22) = 2.08, *p* = 0.0497, Cohen's d = 0.86, a large effect.

**Figure 5:**
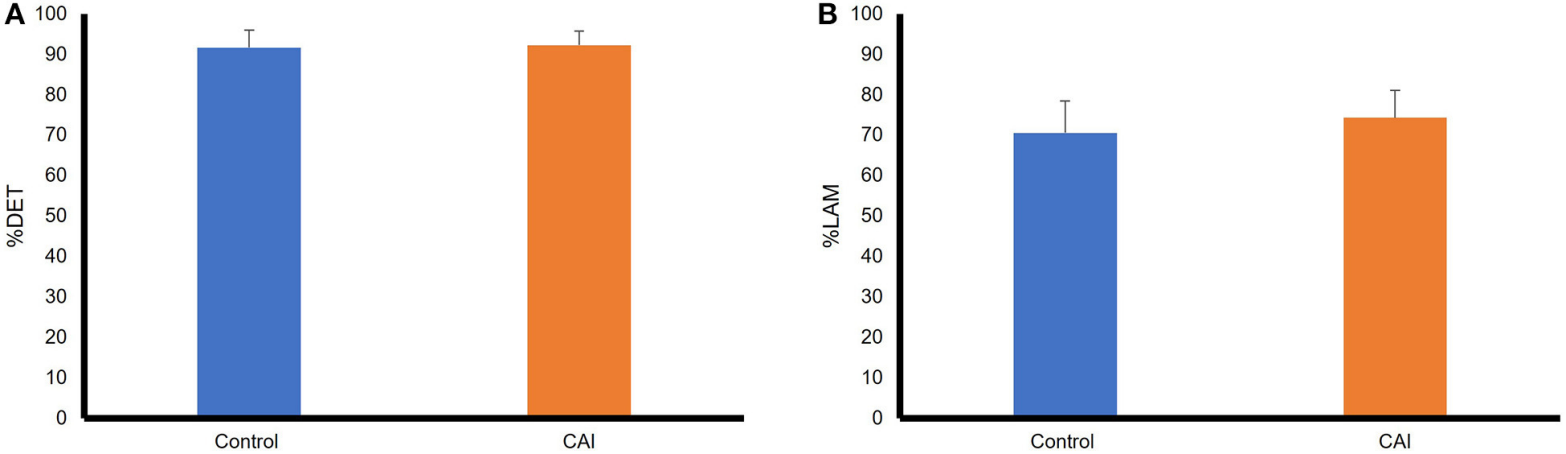
Comparisons of RQA variables between the CAI and control groups in the sagittal plane: **(A)** A comparison of percent determinism (%DET); **(B)** A comparison of percent laminarity (%LAM). The error bar represents the standard deviation. RQA, recurrence quantification analysis; CAI, chronic ankle instability.

Figure 5 summarizes the results of RQA variables in the sagittal plane. The mean %DET was essentially the same between the groups, t(22) = −0.53, *p* = 0.6, Cohen's d = −0.03, a small effect. The %LAM was slightly higher in the control group than that in the CAI group, but the difference did not reach a statistical significance, t(22) = −1.17, *p* = 0.26, Cohen's d = −0.4, a small effect.

## Discussion

We tested the hypothesis that CAI is associated with changes in the ankle dynamics as assessed by measures of determinism (%DET) and laminarity (%LAM) using RQA. This hypothesis was partially supported by the results based on %LAM in the frontal plane. The lower %LAM observed in participants with CAI suggested that their ankle dynamics were less likely to stay in a specific state compared to their healthy counterparts. This more frequent switching between states could reflect compensations for instabilities associated with control at the ankle joint. However, such between-group difference in %LAM was not observed in the sagittal plane. Because ankle sprains or giving ways most often occur in the frontal plane, it is sensible to find deviation in ankle dynamics in the frontal but not the sagittal plane in individuals with CAI. It is important to note that the RQA results were computed from the phase space trajectory, and therefore cannot be mapped directly onto kinematic fluctuations in the original ankle inversion-eversion trajectory. No significant results were found in %DET in both frontal and sagittal planes. This variable indicates whether the ankle dynamics moves through the phase space in a regular, predictable manner. Thus, researchers suggested that this variable may reflect the predictability of a dynamical system (Marwan et al., 2007). Based on our results, the ankle dynamics are similarly predictable between those with and without CAI. Compared to %DET, %LAM seems to be a better variable for screening CAI in a walking task.

The term “state” refers to the location in the phase space in RQA, but its physiological or clinical meaning has not been established previously. To interpret the results of %LAM in the context of CAI, an initial step is to examine whether the results can be linked to symptoms of CAI. A major symptom of chronic ankle instability (CAI) is episodes of the ankle giving way, which has been defined as “the regular occurrence of uncontrolled and unpredictable episodes of excessive inversion of the rear foot, which do not result in an acute lateral ankle sprain” (Gribble et al., 2014). In principle, the likelihood of experiencing ankle giving way will increase when individuals do not (or are not able to) control the ankle motion consistently. CAI involves not only mechanical instability but may also be manifested as a sensorimotor control problem (functional instability). The lower %LAM in individuals with CAI may reflect a more frequent switching between states due to instabilities in neuromotor control processes. In an unstable system, the degrees of freedom are not tightly controlled (Kelso, 1995), which may explain why random giving way would happen in this population. Individuals with CAI have shown deficits in both feedback and feedforward mechanisms in motor control (Gutierrez et al., 2009; Hass et al., 2010; Yen et al., 2016). The feedback mechanism is required in detecting and correcting movement errors, and the feedforward mechanism is required in generating consistent motor outputs. Deficits in the feedback and feedforward mechanisms may provide an explanation for why the ankle dynamics switch between states more often in individuals with CAI.

Some previous studies also showed that the degrees of freedom are more loosely controlled in individuals with CAI compared to those without CAI, as indicated by increased trial-to-trial variability measured by the standard deviation (Brown et al., 2009; Kautzky et al., 2015; McGrath et al., 2017). The increased variability was observed in ankle/foot kinematics or ankle muscle activities during a walking (Kautzky et al., 2015), running (McGrath et al., 2017), or jumping task (Brown et al., 2009). However, contradictory evidence does exist, showing that individuals with CAI demonstrated lower variability in ankle kinematic variables during walking (Herb et al., 2014). Terada et al. (2015) also found a lower stride-to-stride variability in individuals with CAI during treadmill walking using sample entropy, which is another nonlinear method. The sample entropy technique was applied to the original kinematic trajectory, instead of the reconstructed phase space trajectory as used by RQA in this study. Future study is warranted to understand what factors may cause the results to be inconsistent. Potential factors include, but are not limited to, different participant characteristics, tasks, and experiment setups. A more uniform paradigm is needed in the future for examining the association between CAI and the direction of variability change.

Using RQA to analyze ankle dynamics associated with CAI was an innovation of this study. A unique procedure in RQA is using time-delay embedding to reconstruct the original one-dimensional time series to higher-dimensional phase space. This procedure makes the time-dependence of the data a focus of the analysis (von Oertzen and Boker, 2010), and may reveal information hidden or distorted in the original time series. Despite this advantage, the interpretation of results becomes less straightforward in clinical settings. For example, a decrease in %LAM in a five-dimensional space is not observable by qualitative analysis or a simple video analysis, making it difficult for clinicians to use such information for evaluation and intervention. A future study is warranted to link RQA to clinical practice.

Our study had some limitations. Our sample was not balanced in terms of gender. This was because more females responded to our recruitment. We were not aware of previous research that examined the gender effect on motor performance in individuals with CAI. This limitation will be addressed in our follow-up study. The walking tests were performed on the treadmill rather than over the ground, which may affect the generalizability of our results to real-life situation. However, performing the task on the treadmill in a limited lab space allowed us to record sufficient gait cycles for conducting RQA.

## Conclusion

We tested the hypothesis that CAI is associated with changes in the ankle dynamics as assessed by measures of determinism (%DET) and laminarity (%LAM) using RQA. The %LAM was able to detect a change in ankle dynamics in individuals with CAI during walking. This result may reflect more frequent switching between different patterns of neuromuscular control states due to instabilities associated with CAI. With further study and development, this measure may have the potential to become a useful biomarker for CAI.

## Data Availability Statement

The raw data supporting the conclusions of this article will be made available by the authors, without undue reservation.

## Ethics Statement

The studies involving human participants were reviewed and approved by Human Subject Research Protection, Northeastern University. The patients/participants provided their written informed consent to participate in this study.

## Author Contributions

S-CY conceived and designed research. EF collected data. SQ, CH, and C-AC contributed analytical tools. S-CY, EF, SQ, CH, and C-AC analyzed and interpreted data. S-CY drafted the manuscript. All authors read and approved the manuscript.

## Conflict of Interest

The authors declare that the research was conducted in the absence of any commercial or financial relationships that could be construed as a potential conflict of interest.

## Publisher's Note

All claims expressed in this article are solely those of the authors and do not necessarily represent those of their affiliated organizations, or those of the publisher, the editors and the reviewers. Any product that may be evaluated in this article, or claim that may be made by its manufacturer, is not guaranteed or endorsed by the publisher.
